# Beliefs about animal emotions are associated with self-reported consumer behaviors related to animal use

**DOI:** 10.1371/journal.pone.0354794

**Published:** 2026-07-29

**Authors:** Michael A. Kisley

**Affiliations:** Department of Psychology, University of Colorado, Colorado Springs, Colorado, United States of America; Universidade de Sao Paulo Campus de Sao Paulo: Universidade de Sao Paulo, BRAZIL

## Abstract

Human-held beliefs about the emotional capacity and experience of non-human animals appear to be related to attitudes concerning animal use, but direct links between such beliefs and consumer behaviors that affect animals have not been investigated. Two survey-based studies were conducted to test whether scores on the Beliefs about Animal Emotions Scale (BAES) are associated with self-reported consumer behaviors. In Study 1, beliefs about animal emotions statistically predicted reported frequency of non-dietary animal use avoidances (e.g., avoiding animal-tested products, animal-based competitions), over and above age, gender, and educational attainment in a U.S.-based sample. Study 2, which involved targeted recruitment of self-identified vegetarians and vegans, largely replicated these findings, and further showed that BAES scores predicted the number of reported animal-product food avoidances. Having established a link between beliefs about animal emotions and animal-related behavioral choices, additional analyses were conducted to investigate potential factors that contribute to such beliefs. Educational attainment was a consistent negative predictor of BAES scores across both studies: more highly educated participants attributed less emotional capacity and experience to animals. Exploratory analyses indicated that, based on self-report, there are numerous sources of information that contribute to beliefs about animal emotions including, most notably, direct interaction with pets and personal belief systems (e.g., moral, spiritual). Together, these results indicate that individual differences in beliefs about animal emotions, in particular beliefs concerning emotional complexity and authenticity, as well as beliefs about the moral relevance of animal emotions, are importantly related to self-reported consumer behaviors that have implications for animals.

## Introduction

People’s beliefs about non-human animal (hereafter “animal”) psychology have direct and indirect effects on animals. For example, the attribution of *sentience* to animals predicts positive attitudes and behavioral intentions towards a variety of non-human species [1–4]. Notably, this includes beliefs about a subset of internal psychological states, *animal emotions*, as a growing body of work suggests that individual human differences in the attribution of emotion experience and capacity to animals is associated with measurable variation in welfare outcomes for animals [5,6]. Extending past research, this paper describes two studies designed to investigate whether people’s beliefs about animal emotions predict consumer-related behaviors that may affect animals, that is dietary and non-dietary avoidances of animal products. The following sub-sections provide more detail to justify and explain the research approach taken here.

### Connecting beliefs to behaviors

The connection between beliefs about animal emotions and human behaviors that may affect animals can be understood through established frameworks of belief-based action and moral inclusion. In the Theory of Planned Behavior, beliefs about outcomes contribute to attitudes and intentions, which in turn predict behavior in relevant contexts [7]. Consistent with this general model, beliefs more specifically about *emotions* are reliably associated with how individuals appraise emotional situations and select behavioral responses [8,9]. A complementary line of work on moral inclusion indicates that when people believe a target has a rich and complex emotional life, they are more likely to regard that target as part of their moral community, which is associated with greater empathic concern and willingness to help [10]. This logic was originally demonstrated in human–human contexts: for example, beliefs about others’ capacity for “secondary emotions” (e.g., grief, guilt) predict prosocial support [11]. But this same reasoning can be applied to human-animal interaction. In support of this approach, Leach and colleagues [12] found that participants’ perception regarding the capacity of animals to experience complex emotion was among the most impactful for assigning moral status to animals (along with capacity for empathy, suffering, and social bonding), outweighing the contribution of one’s perceptions of animals’ capacity for agency and thinking. Connecting these ideas to form an overarching framework: one’s beliefs about animal emotions inform, in part, their attitudes regarding the moral standing of animals, and therefore their behavior as it may impact animals. The studies described here did not test all components of this model. Motivation, moral or otherwise, was not directly investigated. Rather, the relationship between one’s beliefs about animal emotions and their behavior was the primary focus.

### Measuring beliefs and behaviors

To measure beliefs about animal emotions, the present research employed the Beliefs about Animal Emotions Scale (BAES), a psychometrically validated, multidimensional instrument developed to assess lay beliefs about animals’ emotional capacity and experience [13]. In its initial validation, exploratory factor analysis in a U.S. sample yielded a three-factor, 23-item structure with good internal consistency, comprising subscales that capture beliefs about (i) the complexity and authenticity of animal emotions, (ii) the independence of animal emotions from human similarity, and (iii) the moral relevance of animal emotions. A confirmatory factor analysis in an independent U.K. sample supported this structure [13]. The BAES was explicitly designed to address limitations of prior *ad hoc* animal emotion belief measures that had not undergone factor analysis or validation, thereby preventing cross-study comparability. Association between BAES scores and human behaviors had not previously been tested before the present research.

To quantify dietary practices related to animal use, an adapted Dietarian Identity Questionnaire (adapted DIQ) approach was employed to assess the animal-derived food products that respondents generally avoid [14]. This pattern-based approach was used rather than relying on self-applied dietary labels (e.g., “vegan” or “vegetarian”), which often do not correspond closely to actual consumption practices [15]. Additionally, choices with potential implications for animals extend beyond food consumption, and these behaviors have received less systematic attention across multiple non-dietary domains. To broaden the scope of investigation, an Animal Use Avoidance Index (AUAI) was developed here to assess self-reported avoidance of products and activities involving animals (animal-derived clothing, products tested on animals, animal competitions, etc.).

### Broad overview of current studies

The present research was designed to test whether beliefs about animal emotion predict consumer behaviors that have implications for animals. Self-reported observational data were collected from two independent samples: an open-recruited sample (Study 1) and a targeted sample of self-described vegetarians/vegans (Study 2), both from an online research participant database. Behavioral avoidances, measured by the adapted DIQ and AUAI, were regressed onto select demographic variables and BAES scores, in order to determine if beliefs about animal emotions predict behavior above and beyond any potential demographic effects. Following the theoretical framework outlined above connecting beliefs about animal emotions to behavioral outcomes, and also drawing from past research demonstrating a link between one’s beliefs about the mental and emotional lives of animals and one’s attitudes and behavioral intentions [1,2,4,16], it was predicted here that higher BAES scores would predict more consumer-based behavioral avoidances, both dietary and non-dietary.

In order to begin developing a better understanding of the factors that influence beliefs about animal emotions, two secondary research aims were pursued. First, beliefs about animal emotions as measured by the BAES have not yet been compared to demographic variables, although these have been shown to explain some variation in human beliefs about animal psychology (reviewed below). As such, scores on the BAES subscales were regressed onto age, gender, and educational attainment. Second, an effort was made to explore potential information sources (personal experience with animals, formal and social media, broad belief systems, etc.) that may inform beliefs about animal emotions.

## Study 1 design and predictions

Two separate hierarchical regression analyses were planned to test whether beliefs about animal emotions predict animal-related consumer behaviors above and beyond any potential demographic effects. Dietary (DIQ) and non-dietary (AUAI) consumer behaviors were the outcome variables. Age, gender and education were entered in the first step. Scores on all 3 subscales of the BAES were entered in the second step. In addition to testing whether the step 2 additions significantly increased the amount of variance accounted for by the regression model, individual BAES subscale scores were examined to see if they were significant predictors of behavior. As described in more detail below, and due to an overall lack of sufficient variation in the DIQ scores to support regression analyses (i.e., very few food avoidances were reported across this open-recruitment sample), the hierarchical regression linking beliefs about animal emotions and dietary behaviors was not conducted for this first study.

As a secondary goal, covariation of beliefs about animal emotions with select demographic variables was investigated, as this has not yet been reported for the BAES. Compared to men, women have often been found to endorse stronger beliefs regarding animal sentience [17–19] including attribution of complex or secondary emotions [20–22]. As such, it was predicted that women would exhibit higher scores on the BAES than men here. Concerning adult age, and despite somewhat complex patterns of findings dependent on specific discrete emotional categories investigated [22,23], the relatively few studies that report such effects suggest that advancing age is associated with greater endorsement of belief in animal sentience including but not limited to emotion capacity broadly speaking [4,19,24]. Accordingly, it was predicted here that BAES scores would increase with age. Effects of secondary and post-secondary education on beliefs concerning animal mental and emotional experience have tended to vary based on cultural context. For example, Paul and Podberscek [25] found that belief in animal sentience declined with increasing years of veterinarian study at UK universities (although see [26]). Furnham and Heyes [18] found that advanced UK psychology students attributed less emotion capability to animals than earlier-year students. By contrast, Tamioso and colleagues [19] found the opposite relationship, where more education was associated with greater attribution of mental and emotional capacity in a rural Brazilian sample (Tamioso, personal communication). Because the current study was drawn from a US-based population, and given the greater cultural proximity to the UK compared to Brazil, it was hypothesized here that advancing education would be associated with lower BAES scores.

Finally, an exploratory approach was used to examine the relative frequency of self-reported sources of information that may be important to the development of one’s beliefs about animal emotions (e.g., direct experience with animals, social media, documentary movies). The primary purpose here was to collect preliminary data for an instrument that could be employed in Study 2. Items were inspired by the limited literature available concerning potential sources of beliefs about animal emotions and empathy for animals (e.g., [27,28]), as well as discussion with researchers who study the human-animal relationship in the context of animal emotions and other internal states. No *a priori* hypotheses were formed for this component of the study.

## Study 1 materials and methods

### Participants

Participants were recruited from Prolific.com, an online research pool previously shown to provide strong data quality and more representative demographics compared to other online participant tools and university student populations [29,30]. A total of 400 adults initiated the survey within the U.S. Eight discontinued participation prior to completion and were removed, yielding 392 cases. Of these, five respondents indicated that English was not their primary language and were excluded. Three attention checks were distributed across the survey to further improve data quality; 20 additional respondents failed at least one attention check and were excluded. Following the guidance of Leiner [31], to reduce inclusion of careless responders, submissions completed in less than half the median completion time (461 sec) were removed (an additional 24 participants), leaving a final analytic sample of *N* = 343.

For the final sample (*N* = 343), participants’ mean age was 42.6 years (*SD* = 14.3; range = 18–83). Gender was reported as 160 men (46.6%), 180 women (52.5%), and 3 self-described (2 nonbinary; 1 gender-fluid; 0.9%). Self-identified race/ethnicity was as follows: 12 Asian (3.5%), 71 Black/African American (20.7%), 7 Hispanic/Latino (2.0%), 238 White/Caucasian (69.4%), 3 American Indian/Alaska Native (0.9%), 10 more than one (2.9%), 1 “other—not listed” (0.3%), and 1 who did not respond (0.3%). Educational attainment: 1 without a high-school diploma (0.3%), 39 with a high-school diploma or equivalent (11.4%), 63 with some college (18.4%), 148 with a bachelor’s degree (43.1%), 66 with a master’s degree (19.2%), and 26 with a doctoral degree or equivalent (7.6%). For analyses, educational attainment was collapsed and recoded as highest degree attained: no bachelor-level degree (reference group; *n* = 103), bachelor-level degree highest attainment (*n* = 148), and graduate-level degree highest attainment (*n* = 92). The latter two groups were dummy-coded against the reference group for regressions.

### Measures

*Beliefs about Animal Emotions Scale (BAES;* [13]). The BAES is a factor-analyzed, construct-validated measure of people’s beliefs concerning the emotional lives of animals. Convergent and discriminant validity for the BAES were originally demonstrated through large effect size associations with *Belief in Animal Minds* [32], and small or non-detected associations with social desirability, human-human affective empathy, and one’s tendency to anthropomorphize non-living objects. Criterion validity was established through robust correlations with attitudes towards animal use, specifically scores on the *Animal Attitudes Scale* [33].

Before completing item ratings, participants read the following instructions: “*We are interested in your beliefs about the emotional lives of animals. For animals, you may think of pets, farm animals, wildlife, and other animals. For emotions, you may think of feelings like sadness, happiness, fear, and other feelings. There are no right or wrong answers. We are interested in your personal viewpoint.”* For most items, except 2 that are reversed scored, greater disagreement on a 7-point Likert-type scale (*Strongly disagree*, 7; *Disagree*, 6; *Somewhat disagree*, 5; *Neither agree nor disagree*, 4; *Somewhat agree*, 2, *Agree*, 2, *Strongly agree*, 1) corresponds to greater endorsement of animal emotion function. Scores are computed for 3 subscales: *Animal Emotions Complexity and Authenticity* (11 items, e.g., “An animal’s emotions are basically just simple reactions to threats or rewards”), *Animal Emotions Independence from Human Similarity* (5 items, e.g., “The more similar an animal is to humans, the more rich its emotional life”), and *Moral Relevance of Animal Emotions* (7 items, e.g., “Animals’ emotional states matter less than human emotions”). All subscale reliabilities were reported as good or better across multiple studies, ranging between α = 0.85 and 0.95 in the original description of the scale [13]. Scale reliabilities for the current study were similar, and are provided in Table 1.

**Table 1:** Descriptive Statistics and Reliabilities for Subscale Composites of the Beliefs about Animal Emotions Scale (BAES) for Study 1/Study 2.

BAES Subscale	1	2	3	*M*	*SD*
1. Animal Emotions Complexity and Authenticity	(0.95/0.94)			4.76/4.91	1.40/1.37
2. Animal Emotions Independence from Human Similarity	0.33/0.31	(0.85/0.84)		3.22/3.46	1.18/1.25
3. Moral Relevance of Animal Emotions	0.63/0.65	0.31/0.35	(0.91/0.90)	4.48/4.87	1.46/1.40

*Note*. Each value represents Study 1/Study 2. *N* = 343 for Study 1 and 251 for Study 2. All correlations were significant, *p* < 0.001. Cronbach’s α for each subscale are shown on the diagonal in parentheses.

*Sources of beliefs about animal emotions*. Immediately after the BAES, participants completed an exploratory, single-item measure intended to identify the primary perceived source of their beliefs about animal emotions. The prompt read: *“Which of the following has had the most influence on your beliefs about the emotional lives of animals, specifically about the questions you answered on the previous page?”* Respondents were instructed to select one option from a list—Educational classes (high school, college, etc.); Things my pets do (past and present); Behavior of animals I work with (job, farm, etc.); Documentary movies and TV; Social media posts (TikTok, Facebook, etc.); Fictional stories, folk tales, or cultural stories about animals—with an “Other (please specify)” category permitting a write-in response. Because this instrument was designed for descriptive profiling rather than psychometric scaling, responses were analyzed as a nominal categorical variable to provide a preliminary investigation of self-reported sources of belief.

*Adapted Dietarian Identity Questionnaire (DIQ;* [14]. Dietary choices that impact animals were operationalized using a dietarian‐pattern approach adapted from the DIQ which measures the animal products people generally exclude rather than relying on socially constructed labels such as “vegetarian” or “vegan.” The DIQ framework argues that label-based items oversimplify dynamic food identities, vary cross‐culturally, and often misclassify individuals (e.g., many self-identified vegetarians do not strictly avoid all meats; and ambiguity often exists about whether poultry or fish should be considered as “meat”). Accordingly, it recommends assessing the dietary pattern directly as the combination of red meat, poultry, fish, eggs, and dairy that respondents generally avoid, emphasizing typical efforts rather than absolute abstinence (“never”) to capture overall intentions. In line with this rationale, six adapted items that retained the DIQ’s wording but tailored product categories to study aims were developed. In response to the instructions “*In general, which of the following do you* ***not*** *eat? Please select all that apply,*” participants can select “I generally do not eat beef,” “…foods that are made with cow’s milk,” “…chicken,” “…fish,” “…eggs,” and/or “…pork.” This adaptation preserves the DIQ’s construct validity advantages—granularity across the spectrum of animal-product exclusion and reduced misclassification from labels—while allowing finer resolution within red meat (beef vs. pork) and greater clarity for dairy (cow’s-milk–based foods). A total dietarian identity score was computed for each participant, ranging from 0 to 6, by summing the number of foods they endorsed as generally not eating.

*Animal Use Avoidance Index (AUAI)*. To assess prevalence of additional, non-dietary behaviors that are related to animals, a novel six-item instrument was developed for this study. The following domains were targeted: wild animal exhibitions [34,35], purchase of animal-derived apparel or bedding [36,37], lethal pest control [38], consuming products tested on animals [39,40], and attendance at animal-based competitions [41,42]. Following the DIQ framework and approach described immediately above, items were worded in the first person as habitual avoidances: “I generally avoid killing pest animals like mice”; “… animal-based competitions (rodeos, animal races, livestock shows, etc.)”; “… clothing with animal products (leather, fur, etc.)”; “… places that keep wild animals for exhibition (zoos, marine parks, etc.)”; “… bedding with real bird feathers”; and “… products that were tested on animals.” Items that pertain to pets and hunting were specifically avoided because it has been found that people often endorse highly conflicting viewpoints with regard to these animal groups [43,44]. For this study, responses were aggregated to provide a summed avoidance count (0–6) for each participant. Instructions for this index read “In general, which of the following do you avoid? Please select all that apply.”

### Procedure

This research protocol was approved by the IRB of the University of Colorado – Colorado Springs. Human participants provided written informed consent. Data collection occurred on 17/03/2025. Participants self-selected into the study on Prolific.com. After providing informed consent, they completed the BAES, the belief sources questions, the adapted DIQ, the novel AUAI, and finally answered demographic questions. Each was compensated $1.50 US.

## Study 1 results

### BAES descriptive variables

Descriptive statistics and intercorrelations for the BAES subscales are summarized in Table 1 and were found to be comparable to previously published values [13].

### Relationship between beliefs about animal emotions and dietary-related behaviors that impact animals

The distribution of total adapted DIQ scores (from 0 to 6 dietary avoidances) did not exhibit normality (*M* = 0.71, *SD* = 1.25, skewness = 2.37; kurtosis = 5.80), reflecting a strong floor effect and very few responses at the upper end. Most respondents reported no exclusions across the six food categories (216 of 343, 63.0%); 21.6% avoided one category, 7.0% avoided two, 3.8% avoided three, 1.2% avoided four, 2.0% avoided five, and 1.5% avoided all six (item level responses rates are provided in Table 2). As such, very few participants in this sample reported any systematic avoidance of food products, and a majority reported no avoidances at all. Most who did only avoided 1 food group, which may represent factors other than animal-impact motivated choices such as food allergies or religious observances, for example.

**Table 2:** Item-Level Response Rates to Dietarian Identity and Animal Use Avoidance Behavior Measures, Studies 1 and 2.

Item	Study 1		Study 2	
	#	%	#	%
**Adapted DIQ**				
I generally do not eat beef	44	12.8%	131	52.2%
…pork	87	25.4%	158	62.9%
…chicken	19	5.5%	112	44.6%
…fish	46	13.4%	106	42.2%
…eggs	18	5.2%	62	24.7%
…foods that are made with cow’s milk	28	8.2%	60	23.9%
**AUAI**				
I generally avoid killing pest animals like mice	112	32.7%	125	49.8%
…products that were tested on animals	123	35.9%	122	48.6%
…clothing with animal products	73	21.3%	101	40.2%
…places that keep wild animals for exhibition	63	18.4%	76	30.3%
…bedding with real bird feathers	111	32.4%	114	45.4%
…animal-based competitions	103	30.0%	112	44.6%

*Note*. For Study 1, *N* = 343 from open recruitment on Prolific.com. For Study 2, *N* = 251 from targeted recruitment for self-described vegans and vegetarians. *Adapted DIQ*, Adapted Dietarian Identity Questionnaire. *AUAI*, Animal Use Avoidance Index. *#,* number of participants that endorsed each item; *%,* percentage of the sample that endorsed each item.

Given the highly restricted and non-normal distribution of reported dietary-related behaviors, regression analyses connecting beliefs to these behaviors were not conducted for this sample. Instead, this result motivated a more targeted recruitment strategy for Study 2 in the hopes of obtaining a broader distribution of dietarian identity scores.

### Relationship between beliefs about animal emotions and other (non-dietary) behavioral avoidances

The distribution of total AUAI scores (from 0 to 6 avoidances) was relatively normal based on examination of sample statistics (*M* = 1.71, *SD* = 1.73, skewness = 0.97, kurtosis = 0.09), though nevertheless concentrated in the direction of lower values: 31.5% (108 of 343) endorsing no avoidances; 24.2% endorsing one, 16.9% two, 12.0% three, 5.8% four, 4.4% five, and 5.2% avoiding all six behaviors (item level responses are provided in Table 2). A majority of respondents (68.5%) reported at least one avoidance. Given the reasonably broad distribution of values and statistical evidence of normality for the AUAI, it was decided that regression analyses connecting this measure to beliefs about animal emotions could be conducted as planned.

Reported prevalence of non-dietary animal-related consumer behaviors was predicted through regression in two steps: first by demographic variables alone, and in a second step by adding all 3 subscales of the BAES to the model. In this way, it was possible to test whether people’s beliefs about animal emotions provided predictive power above and beyond their demographic characteristics. The initial model with only demographic variables as predictors was significant, *F*(4,335) = 6.730, *p* < 0.001, accounting for 6.3% of variance in AUAI scores (*R*^*2*^ = 0.063). All regression coefficients and corresponding error estimates can be found in Table 3. Adding the 3 BAES subscales significantly increased the amount of variance predicted by the model, Δ*F*(3,333) = 19.373, *p* < 0.001, Δ*R*^*2*^ = 0.138. This full model was significant, *F*(7,332) = 12.781, *p* < 0.001, and explained about 20% of the variance in AUAI scores (*R*^*2*^ = 0.196). Of the demographic variables, age was the only significant predictor, in a positive direction (i.e., higher age was associated with more behavioral avoidances). All 3 subscales of the BAES were significant predictors, such that higher endorsement of beliefs in animal emotion function was associated with more reported behavioral avoidances that are related to animal use.

**Table 3:** Coefficients for Non-Dietary Avoidances Regressed on Demographics and Beliefs About Animal Emotions – Studies 1 and 2.

Variable	Study 1			Study 2		
	*B*	*SE B*	β	*B*	*SE B*	β
Step1						
Constant	0.175	0.401		2.575***	0.628	
Age	0.021**	0.006	**0.177**	0.018	0.010	0.112
Gender	0.554**	0.182	**0.162**	0.349	0.258	0.081
Bachelors	−0.207	0.215	−0.060	−1.197***	0.349	**−0.283**
Graduate	−0.576***	0.239	**−0.150**	−1.924***	0.343	**−0.458**
Step2						
Constant	−2.046***	0.478		−1.286	0.779	
Age	0.021***	0.006	**0.173**	0.012	0.009	0.073
Gender	0.311	0.173	0.091	0.258	0.233	0.060
Bachelors	−0.088	0.201	−0.025	−0.513	0.323	−0.121
Graduate	−0.406	0.225	−0.106	−1.008**	0.332	**−0.240**
BAES 1	0.166*	0.081	**0.136**	0.534***	0.109	**0.351**
BAES 2	0.201**	0.075	**0.139**	−0.054	0.098	−0.032
BAES 3	0.242**	0.076	**0.206**	0.243*	0.108	**0.162**

*Note*. Criterion (outcome) variable for this analysis was total Animal Use Avoidance Index (AUAI; range 0–6). For Study 1, *N* = 340 (only Man and Woman genders) from open recruitment on Prolific.com. For Study 2, *N* = 247 (only Man and Woman genders) with targeted recruitment for vegans and vegetarians. *Bachelors*, highest degree attained bachelors or equivalent compared to no college degree. *Graduate*, highest degree attained graduate-level compared to no college degree. *BAES 1*, beliefs about animal emotion complexity and authenticity; *BAES 2*, beliefs regarding independence of animal emotions from human similarity; *BAES 3*, beliefs about moral relevance of animal emotions. Interpretable standardized β-weights are bolded for emphasis and to facilitate comparisons between Studies 1 and 2.

*p < 0.05; **p < 0.01; ***p < 0.001.

To summarize, and consistent with *a priori* hypothesis, each of the 3 subscales of the BAES uniquely predicted non-dietary avoidances that are related to animals, and did so above and beyond one’s age, gender, and education. Higher endorsement of animal emotion complexity and authenticity, animal emotion independence from human similarity, and moral relevance of animal emotion *each separately* predicted the frequency of these animal-relevant behaviors.

### Demographic effects on beliefs about animal emotions

To investigate the relationship between beliefs about animal emotions and demographic variables, each BAES subscale was regressed on age, gender (coded 1 = man, 2 = woman; self-described/nonbinary respondents excluded to preserve this coding, n = 3), and education (highest degree attained: bachelors degree or equivalent compared to no college degree reference; graduate-level degree compared to no college degree reference). Key findings are reported immediately below, and full regression model results are provided in Supplemental Materials Table S1 in S1 File.

Demographic variables were found to predict beliefs about animal emotions. The overall regression model for subscale 1, *Animals Emotion Complexity and Authenticity*, was significant, *F*(4, 335) = 5.011, *p* < 0.001, with demographics explaining 5.6% of the variance (*R*^*2*^ = 0.056). Age was a significant positive predictor, *B* = 0.013, *SE* = 0.005, β = 0.129, *t*(336) = 2.391, *p* = 0.017, as was gender, *B* = 0.296, *SE* = 0.150, β = 0.106, *t*(336) = 1.980, *p* = 0.048. Tha*t* is, increasing age and being a woman predicted greater endorsement of animal emotion complexity and authenticity. Highest educational attainment of bachelors degree, *B* = −0.389, *SE* = 0.177, β = −0.138, *t*(336) = −2.195, *p =* 0.029, and gradua*t*e degree, *B* = −0.610, *SE* = 0.197, β = −0.194, *t*(336) = −3.096, *p* = 0.002, were bo*t*h significant negative predictors. In other words, increasing education predicted less endorsement. The model for subscale 2, *Animal Emotions Independence from Human Similarity*, was not significant, *F*(4, 335) = 1.273, *p* = 0.456, *R*^*2*^ = 0.011. The model for subscale 3, *Moral Relevance of Animal Emotions*, was significant, *F*(4, 335) = 4.419, *p* = 0.003, with demographics accounting for 4.7% of the variance (*R*^*2*^ = 0.047). Gender was the only significant individual-level predictor for this subscale, *B* = 0.633, *SE* = 0.157, β = 0.217*, t*(336) = 4.030, *p* < 0.001, with women endorsing greater moral relevance of animal emotions than men.

In summary, all 3 demographic variables studied–age, gender, and education–were found to be significant predictors of some aspect of beliefs about animal emotions, and in the directions hypothesized based on past literature. That being said, the amount of variance accounted for was relatively small: total variance in beliefs about animal emotions accounted for by demographic variables was 5–6% or less depending on the particular subscale.

### Sources of beliefs about animal emotions – exploratory

Participants’ attributions for the primary source of their beliefs about animal emotions spanned all options, with the highest proportion for “Things my pets do (past and present)” at nearly 50% (*n* = 170 of 343 total, 49.6%), and next highest for “Documentary movies and TV” at 20.7% (*n* = 71). All other sources were endorsed at a rate less than 10% (response rates are provided in Supplemental Table S2 in S1 File). Just over 6% of participants (*n* = 21) endorsed “Other,” and correspondingly provided write-in explanations. Recurring themes referenced personal belief systems (both religious and secular), independent reading, and podcasts. Notably, a subset used Other to describe multiple influences despite the single-choice instruction, suggesting that the forced-choice format may have constrained some respondents’ ability to represent their beliefs accurately. All narrative responses are reproduced verbatim in the Supplementary Materials (Table S3 in S1 File).

No inferential analyses were conducted on the source-of-beliefs item because it was designed as an exploratory, single forced-choice nominal indicator of a multifactorial construct. Also, several respondents explicitly used “Other” to list multiple influences, underscoring the likely mismatch between the response format and the underlying construct. Contrasts of BAES scores between or within specific categories (e.g., comparing BAES scores between those who did and did not select a given option) would be difficult to interpret given unequal cell sizes and overlapping influences. Instead, the results of this exploratory measure were used to design an updated assessment for Study 2.

## Study 2 design and predictions

To address design limitations observed in Study 1, Study 2 incorporated two refinements. First, in order to improve the distribution of dietarian-identity scores used to investigate the prevalence of behaviors that impact animals, self-identified vegetarians and vegans were targeted for recruitment. Following the same logic of study 1, it was hypothesized that stronger beliefs concerning emotional capacity of animals would predict greater numbers of avoidances of both food and non-food items and activities that are related to animals in this sample. There was no *a priori* reason to modify hypotheses based on the anticipated difference in sample composition from Study 1.

For the second refinement, the “sources of belief” measure was modified from a single forced-choice item to a comparative format, permitting rating of multiple influences, to enable direct between-source contrasts. This approach was intended to allow for investigation of whether stronger endorsement of certain sources of beliefs is predictive of scores on the BAES (methodological detail is provided below, in Study 2 Materials and Methods). For example, is endorsement of certain sources of beliefs associated with greater/lesser beliefs regarding the emotional capacity of animals? This was tested by regressing BAES scores onto the endorsement ratings for all included information sources. The option set was also revised: categories with very low endorsement in Study 1 were removed and frequently mentioned “Other” themes—personal belief systems (religious and secular), independent reading, and podcasts—were added. Based on past research, experience with pets is associated with greater attribution of sentience and emotional capacity and experience [23,45,46]. As such, it was hypothesized that direct, live observation of pet behavior would predict stronger beliefs concerning the emotional lives of animals. No other specific hypotheses were formulated for this exploratory research.

## Study 2 materials and methods

### Participants

Participants were recruited on Prolific.com with the same procedures as Study 1, but with the addition of a dietary-preference prescreening filter to selectively allow individuals who had previously self-identified as vegan or vegetarian when completing their Prolific profiles. Although sample size was originally planned for 400 (to mirror Study 1), recruitment was found to be very protracted, presumably due to the low number of Prolific.com users who endorsed one of these identities. As such, recruitment was ended once 300 participants were enrolled, at which point it appeared that very few additional viable participants who met the prescreening filter were available. Five respondents who reported that English was not their primary language were excluded, as were an additional 24 who failed one or more attention checks, and a further 20 who completed the survey in less than half the sample median completion time (439 s). The final analytic sample was composed of *N* = 251 individuals, mean age 38.0 years (*SD* = 12.8; range = 18–75). This sample included 91 men (36.3%), 156 women (62.2%), and 4 self-described (1 nonbinary, 1 gender-fluid, 2 transgender; 1.6%). Self-identified race/ethnicity was 6 Asian (2.4%), 57 Black/African American (22.7%), 9 Hispanic/Latino (3.6%), 170 White/Caucasian (67.7%), 2 American Indian/Alaska Native (0.8%), 6 more than one race (2.4%), and 1 “other—not listed” (0.4%). Educational attainment was 0 without a high-school diploma, 13 high-school diploma or equivalent (5.2%), 35 some college (13.9%), 100 bachelor’s degree (39.8%), 78 master’s degree (31.1%), and 25 doctoral degree or equivalent (10.0%). For analyses, educational attainment was collapsed and recoded as highest degree attained: no bachelor-level degree (reference group; *n* = 48), bachelor-level degree highest attainment (*n* = 100), and graduate-level degree highest attainment (*n* = 103). The latter two groups were dummy-coded against the reference group for regressions.

### Measures

*Beliefs about Animal Emotions Scale (BAES).* This scale was administered and scored as in Study 1, where it is described in more detail. All subscale reliabilities obtained in this sample were good or better and are reported in Table 1.

*Updated sources of beliefs about animal emotions instrument*. Immediately after the BAES, participants rated the extent to which each of eight potential sources influenced the beliefs they had just reported (“To what extent has each of the following influenced your beliefs about the emotional lives of animals, specifically about the questions you answered on the previous page?”). Each source was rated on a 3-point Likert-type scale: *Not at all (1), Somewhat (2), A lot (3).* The sources were: Documentary movies and TV; Social media posts (TikTok, Facebook, etc.); Direct, live experience observing pets; Direct, live experience observing animals I work with (job, farm, etc.); Educational classes (high school, college, etc.); Reading books or articles outside of a school setting; Podcasts; and My spiritual, moral, ethical, philosophical, or other belief systems. This list and format were revised from Study 1 to enable comparative assessment across multiple influences and to incorporate categories suggested by Study 1 narrative responses. Higher scores indicate greater perceived influence.

*Consumer behaviors that impact animals*. The adapted DIQ (dietarian-identity) and AUAI (non-dietary avoidances) instruments described for Study 1 were employed here, unmodified.

### Procedure

This research protocol was approved by the IRB of the University of Colorado – Colorado Springs. Human participants provided written informed consent. Data collection occurred from 28/05/2025–29/05/2025. Participants who had self-identified as vegan or vegetarian on their Prolific.com profile self-selected into the study. After providing informed consent, participants completed the BAES, the belief sources items, the adapted DIQ, the AUAI, and then demographic questions. Each was compensated $1.50 US.

## Study 2 results

### BAES descriptive variables

Descriptive statistics and zero‐order correlations for BAES subscale composites obtained for this sample are reported in Table 1. The overall pattern closely parallels Study 1 and previously reported values [13].

### Relationship between beliefs about animal emotions and dietary-related avoidances

The distribution of total adapted DIQ scores (from 0 to 6 dietary avoidances) was relatively normal (*M* = 2.51, *SD* = 2.24, skewness = 0.29; kurtosis = −1.36), with a somewhat broader distribution of responses compared to Study 1: 30.7% (77 of 251) reported no exclusions, 12.7% one, 8.4% two, 10.0% three, 17.1% four, 4.0% five, and 17.1% reported generally not eating all 6 animal products (item level responses are provided in Table 2). As such, the relationship between this variable and beliefs about animal emotions was conducted as planned.

Total count for modified DIQ was modelled using a regression approach. Demographic variables were entered in step 1, and the three BAES subscales were added in Step 2 (full model results are provided in Table 4). This allowed for the evaluation of incremental predictive value of beliefs about animal emotions above and beyond demographics. The step 1 demographics-only model was significant, *F*(4,242) = 6.731, *p* < 0.001, *R*^*2*^ = 0.100. Addition of the 3 BAES subscales significantly improved the model, Δ*F*(3,239) = 18.719, *p* < 0.001, Δ*R*^*2*^ = 0.171, and the full model was significant, *F*(7,239) = 12.714*, p* < 0.001, *R*^*2*^ = 0.271. The only significant individual predictors in this full model were age and BAES subscale 1. Notably, the latter represented a medium effect size (β = 0.396).

**Table 4:** Coefficients for Dietary-Related Behaviors Regressed on Demographics and Beliefs About Animal Emotions – Study 2.

Variable	*B*	*SE B*	β	*t*	*p*	*R* ^*2*^ */ΔR* ^*2*^
Step1						0.100***/0.100***
Constant	2.534***	0.694		3.650	<.001	
Age	0.030**	0.011	**0.173**	2.801	.006	
Gender	−0.025	0.285	−0.005	−0.087	.931	
Bachelors	−1.417***	0.385	**−0.310**	−3.677	<.001	
Graduate	−1.421***	0.379	**−0.313**	−3.753	<.001	
Step2						0.271***/0.171***
Constant	−1.694	0.874		−1.938	0.054	
Age	0.022*	0.010	**0.127**	2.245	0.026	
Gender	−0.060	0.261	−0.013	−0.231	0.818	
Bachelors	−0.694	0.363	−0.152	−1.914	0.057	
Graduate	−0.421	0.373	−0.093	−1.129	0.260	
BAES 1	0.650***	0.122	**0.396**	5.326	<0.001	
BAES 2	0.101	0.110	0.055	0.923	0.357	
BAES 3	0.075	0.121	0.046	0.622	0.535	

*Note*. Criterion (outcome) variable for this analysis was total modified Dietarian Identity Questionnaire (DIQ, range 0–6). Study 2 sample: *N* = 247 (only Man and Woman genders) with targeted recruitment for vegans and vegetarians. *Bachelors*, highest degree attained bachelors or equivalent compared to no college degree. *Graduate*, highest degree attained graduate-level compared to no college degree. *BAES 1*, beliefs about animal emotion complexity and authenticity; *BAES 2*, beliefs regarding independence of animal emotions from human similarity; *BAES 3*, beliefs about moral relevance of animal emotions. Interpretable standardized beta-weights are bolded for emphasis. *p < 0.05; **p < 0.01; ***p < 0.001.

In summary, as hypothesized, beliefs about the complexity and authenticity of animal emotions predicted the total dietarian identity score above and beyond demographics, such that stronger beliefs predicted more animal food product avoidances. Collectively, the model based on age, gender, education and all 3 aspects of beliefs about animal emotions captured by the BAES accounted for just over 25% of variance in dietarian identity.

### Relationship between beliefs about animal emotions and other (non-dietary) avoidances

The distribution of total AUAI scores (from 0 to 6 avoidances) was relatively normal (*M* = 2.59, *SD* = 2.09, skewness = 0.28, kurtosis = −1.27) with 21.9% (55 of 251) endorsing no avoidances; 16.7% endorsing one, 14.3% two, 12.4% three, 9.6% four, 12.4% five, and 12.7% avoiding all six behaviors (item level responses can be found in Table 2). Given the reasonably broad distribution of values, regression analyses connecting this measure to beliefs about animal emotions was undertaken as planned.

Beliefs about animal emotions were found to predict self-reported animal-relevant avoidances above and beyond demographics. Regression coefficients and errors can be found in Table 3. A first step regression model including only demographics (age, gender, and education) was significant, *F*(4,242) = 9.609*, p* < 0.001, *R*^*2*^ = 0.137. Educational attainment variables were the only significant predictors, such that more education was associated with fewer reported avoidances. In a second step, the addition of the 3 subscales of the BAES significantly improved the regression model, *ΔF* (3,239) = 21.774, *p* < 0.001, Δ*R*^*2*^ = 0.185. The overall model was significant, *F*(7,239) = 16.237, *p* < 0.001, *R*^*2*^ = 0.322. Graduate (but not bachelors) degree as highest attainment remained a significant individual predictor. Of the BAES subscales, the first, *Animal Emotions Complexity and Authenticity*, and the third, *Moral Relevance of Animal Emotions*, were significant individual predictors such that stronger endorsement of animal emotions predicted more behavioral avoidances. Beliefs about animal emotions complexity and authenticity predicted non-dietary avoidances with a medium effect size (β = 0.351).

To summarize, beliefs about the complexity and authenticity of animal emotions and beliefs about the moral relevance of animal emotions each uniquely predicted the number of behavioral avoidances that impact animals, and they did so above and beyond demographic effects. For the full regression model that included demographics and all 3 subscales of the BAES, over 30% of variance in self-reported avoidances was accounted for. This pattern of results is consistent with study hypotheses.

### Demographic effects on beliefs about animal emotions

As in Study 1, demographic covariation was investigated by regressing each BAES subscale on age, gender, and education. Full regression results are available in Supplemental Materials Table S4 in S1 File. The model predicting scores on subscale 1, *Animals Emotion Complexity and Authenticity*, was significant, *F*(4,242) = 8.689, *p* < 0.001, *R*^*2*^ = 0.126. Examining individual predictors, both education variables were significant: highest degree bachelors compared to no college degree, *B* = −0.945, *SE* = 0.231, β = −0.340, *t*(243) = −4.093, *p* < 0.001, and highes*t* degree graduate-level compared to no college degree, *B* = −1.258, *SE* = 0.227, β = −0.455, *t*(243) = −5.544, *p* < 0.001. As in Study 1, more educa*t*ion was associated with lower endorsement of animal emotion complexity and authenticity. For BAES subscale 2, *Animal Emotion Independence from Human Similarity*, the overall demographic model was significant, *F*(4,242) = 5.450, *p* < 0.001, *R*^*2*^ = 0.083. As for subscale 1, educational variables were the only significant predictors in this model: bachelors, *B* = −0.444, *SE* = 0.213, β = −0.177, *t*(243) = −2.082, *p =* 0.038; graduate-level, *B* = −0.899, *SE* = 0.210, β = −0.361, *t*(243) = −4.292, *p* < 0.001. The model for BAES subscale 3, *Moral Relevance of Animal Emotions*, was also significan*t*, *F*(4,242) = 8.172*, p* < 0.001, *R*^*2*^ = 0.119. Again, education were the only significant individual predictors: bachelors, *B* = −0.838, *SE* = 0.236, β = −0.296, *t*(243) = −3.555, *p* < 0.001; graduate-level, *B* = −1.210, *SE* = 0.232, β = −0.430, *t*(243) = −5.222, *p* < 0.001.

To summarize, like the results of Study 1, the amount of total variance in beliefs about animal emotions explained by demographics was relatively small, about 10% or less for this sample of participants who were targeted for self-identifying as vegan or vegetarian on Prolific. Unlike the previous study, for which an open recruitment strategy was used, here only education emerged as a significant individual-level predictor variable for all 3 subscales of the BAES. Like Study 1, more education was associated with less endorsement of beliefs about animal emotion function.

### Sources of beliefs about animal emotions

**Fig 1:**
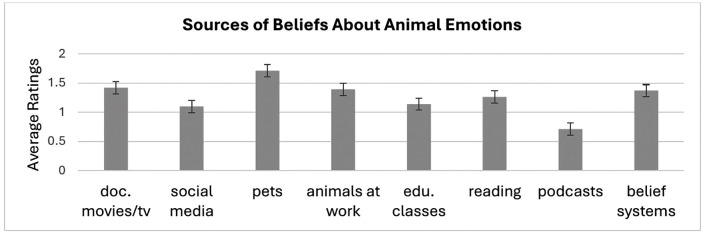
Average endorsement ratings for information sources that inform beliefs about animal emotions, Study 2. Rating options: *not at all* (0), *somewhat* (1), *a lot* (2). Error bars indicate standard errors.

The self-reported relevance of 8 sources of beliefs about animal emotions was investigated through repeated measures ANOVA. Average endorsement rates are shown in Fig 1. Mauchly’s test of sphericity was significant, χ^2^(27) = 148.505, *p* < 0.001. Therefore, the degrees of freedom were corrected using Greenhouse-Geisser estimates (ε = 0.858). There was a significant effect of belief source, *F*(6.006,1501.513) = 56.745, *p* < 0.001. “Direct, live experience observing pets” was the highest rated source, significantly greater than all other sources based on post hoc, Bonferroni-adjusted comparisons (all *p*’s < 0.001). “Podcasts” was numerically the lowest rated sources, significantly smaller than all other sources (all *p*’s < 0.001).

To investigate whether and how these 8 sources of information relate to beliefs about animal emotions, regression analyses were conducted for each of the subscales of the BAES. Demographics were included as covariates, since it was found above that these variables do predict some variance in beliefs about animal emotions.

Self-reported influence of information sources predicted emotion beliefs even when demographic variables were included (full regression model results included in Supplemental materials Table S5 in S1 File). For subscale 1, *Animal Emotions Complexity and Authenticity*, the overall model was significant, *F*(12,234) = 8.511, *p* < 0.001. Approximately 30% of the variance in this subscale was accounted for by this model (*R*^*2*^ = 0.304). Of the sources, direct observation of pets (*B* = 0.457, *SE* = 0.181, β = 0.163, *t*(235) = 2.531, *p* = 0.012), and personal belief sys*t*em (*B* = 0.424, *SE* = 0.115, β = 0.219, *t*(235) = 3.697, *p* < 0.001) were significan*t* positive predictors, and podcasts (*B* = −0.535, *SE* = 0.130, β = −0.268, *t*(235) = −4.117, *p* < 0.001) was a significant nega*t*ive predictor. For subscale 2, *Animal Emotions Independence from Human Similarity*, the regression model was significant, *F*(12,234) = 2.554, *p* = 0.003, *R*^*2*^ = 0.116. No self-reported belief source ratings were significant as individual predictors in this model. For subscale 3, *Moral Relevance of Animal Emotions*, the model was significant, *F*(12,234) = 5.181, *p* < 0.001, *R*^*2*^ = 0.210. The only significant non-demographic individual level predictors in this model were both positive: direct observation of pets (*B* = 0.439, *SE* = 0.196, β = 0.154, *t*(235) = 2.241, *p* = 0.026) and personal belief system (*B* = 0.448, *SE* = 0.124, β = 0.227, *t*(235) = 3.611, *p* < 0.001).

To summarize these results, many different sources of information contribute to one’s beliefs about animal emotions based on self-report. Numerically, all sources except “Podcasts” showed an average rating above 1, which places their relative influence somewhere between “somewhat” and “a lot.” Direct, live observation of pets was rated highest. In terms of whether and how these source endorsements relate to one’s beliefs as measured by the BAES, only pet observation and personal belief system were found to consistently predict beliefs in this sample. More specifically, those who cited these sources as more influential also endorsed stronger beliefs concerning animal emotion complexity and authenticity, as well as the moral relevance of animal emotions. The connection between beliefs about animal emotions and previous experience with pets was consistent with a priori hypothesis.

## General discussion

Based on the 2 studies described here, and in support of hypotheses, individual differences in belief about the emotional capacity and experience of animals were associated with self-reported consumer behaviors that are related to animals. In Study 2, scores on the BAES, in combination with specific demographics (age, gender, educational attainment), collectively predicted just over 25% of variance in self-reported animal-based food product avoidances. In particular, beliefs about the complexity and authenticity of animal emotions (BAES subscale 1) exhibited a medium effect size (standardized β = 0.396) in predicting animal product food avoidances. This is consistent with the research of Bilewicz and colleagues [16] who found that self-described vegetarians ascribed more capacity for “secondary” emotions (e.g., guilt and regret) to animals than omnivores, but they did not detect a difference between groups for “primary” emotions (e.g., fear and happiness).

Beliefs about animal emotions were also associated with other reported consumer behaviors that are related to animals. Combined with demographics, inclusion of all 3 subscales of the BAES explained between 20% (Study 1) and 30% (Study 2) of variance in non-dietary avoidances (e.g., lethal pest control, purchasing animal-based products, and patronizing venues that showcase animals). Beliefs about the complexity and authenticity of animal emotions (BAES subscale 1) and beliefs about the moral relevance of animal emotions (BAES subscale 3) were consistent positive statistical predictors of these reported behavioral avoidances. Beliefs concerning animal emotions’ independence from human similarity (BAES 2) emerged as a unique predictor only in Study 1. One possibility for this pattern is that human similarity serves as a more important basis for animal-related consumer decisions in the general population (open recruitment for Study 1) than among people already inclined toward vegetarian or vegan identity (targeted recruitment for Study 2). Within this more animal-use-conscious sample, avoidance may depend more directly on whether animal emotions are viewed as complex, authentic, and morally relevant, regardless of whether animals’ emotional capacities resemble those of humans. This possibility warrants more direct investigation in future research.

Collectively, these findings provide evidence for a potentially important relationship between one’s beliefs about animal emotions and one’s self-reported behavior in the marketplace, both dietary and non-dietary. This adds to and is consistent with previous research that has shown a connection between beliefs about animal sentience and attitudes regarding animal use [1–4]. Including non-dietary avoidances here broadened the scope of the present research beyond food-related choices alone. Beliefs concerning the complexity and authenticity of animal emotions were associated with both dietary avoidances in Study 2 and non-dietary avoidances across both studies, suggesting that this dimension may be relevant across multiple forms of animal-use-related consumer behavior. Concerning any potential difference in the pattern of relationship between beliefs about animal emotions and consumer behaviors across dietary and non-dietary dimensions, it is important to note that these studies were not designed to compare the different forms of behavior directly. As such, differences in the pattern of predictors should be interpreted with caution, and investigated more explicitly in future research (e.g., through multivariate research designs).

It’s important to note that the regression models for this research were constructed with beliefs about animal emotion as predictors and self-reported consumer behavioral avoidances as outcome variables, following the theoretical framework advanced in the introduction. But additional empirical research will be required to confirm a potential causative relationship, including tests of directionality. The statistical results from these studies are not inconsistent with a model whereby behaviors influence beliefs, as opposed to the other way around. Indeed, it has been shown that meat eating behavior can motivate people to deny animal minds [47,48]. Perhaps an even more likely model involves bidirectional effects between beliefs about animal emotions and animal-related consumer behaviors, that is a co-reinforcing system. Experimental designs in future work are anticipated to be helpful for better understanding this relationship.

The studies described here were designed around the foundational psychological premise that beliefs can shape behavior regardless of their objective accuracy [7,49]. As such, alignment of people’s beliefs about animal emotions with expert consensus was not investigated here. However, the scientific status of animal emotions is relevant for policy and education efforts. Convergent behavioral, physiological, and cognitive evidence increasingly supports the claim that many nonhuman animals possess emotion-like states [50,51], and provides methodology for assessing and comparing these across species [52,53]. Science-backed communication about animal emotion may therefore represent a promising mechanism for indirectly reducing negative impacts on animals, by shifting public perception and behavior. For example, the European Union AFFECT-EVO Cost Action brings together natural scientists, animal welfare scientists, social scientists, and applied fields such as policy and law to develop and disseminate scientific consensus regarding cross-species emotion research (https://affect-evo.eu/). Such initiatives aim to narrow gaps between research and public understanding while carefully avoiding non-scientifically biased attributions, both anthropomorphism and anthropodenial of animals’ emotional capacities [12,50,51].

### What contributes to one’s beliefs about animal emotions?

Having established a link between beliefs about animal emotions and reported animal-relevant consumer behaviors, it is important to better understand the source(s) of such beliefs. This was a secondary aim of the research described here. Results added to past research that has shown there are demographic-based differences in beliefs about animal psychology, although the effects were relatively small and inconsistent here (total demographic effects typically explained between 5% and 10% of variance in beliefs depending on the study and the BAES subscale of interest). Age and gender effects were present, but detection differed across BAES subscales and study sample characteristics (open recruitment for study 1 compared to targeted vegan/vegetarian recruitment for study 2). Increasing adult age was associated with more endorsement of the complexity and authenticity of animal emotions (BAES subscale 1), whereas being a woman predicted stronger beliefs concerning the moral relevance of animal emotions (BAES subscale 3). Overall, this is largely consistent with past research concerning beliefs about animal emotion and, more broadly, animal sentience [4,17,19,21,22].

The most consistent demographic effect observed here was that higher educational attainment predicted beliefs about animal emotions. Having attained a bachelors as highest degree was a significant *negative* predictor of beliefs about animal emotion complexity and authenticity (BAES subscale 1) in both studies, and for beliefs about the moral relevance of animal emotions (BAES 3) in Study 2. Attaining a graduate-level degree was also a significant negative predictor for BAES 1 in Study 1, and all three BAES subscales in Study 2. Collectively, these findings imply that post-secondary education is associated with *less* attribution of emotion function and experience to animals, at least in the U.S.-based, online research platform samples studied here. This is consistent with research on animal sentience in both undergraduate [18] and professional [54] settings in the U.K (but see [26]. At present, it is unknown why more and, apparently, more specialized education leads people to adopt more skeptical beliefs concerning animal minds and internal psychological states. This may be at least partly explained by the finding that animal behavior researchers in higher education settings, and who are therefore in a position to influence student viewpoints, may collectively possess a “conservative bias” when it comes to attribution of emotional states to non-human animals [51].

Preliminary, exploratory research was also conducted here to begin investigating other sources of information that may influence one’s beliefs about animal emotions. Based on self-report, it appears that multiple sources may contribute to the development of such beliefs. Of the sources available for endorsement in Study 2, direct, live experience with pets was the most highly rated. But documentary movies and tv, social media, animals encountered through work, educational classes, additional reading, and personal beliefs systems were all endorsed at an average rate above “somewhat.” Further, it was found that the relative endorsement of certain sources differentially predicted one’s beliefs about animal emotions. Specifically, stronger self-report of direct observation of pets as a source of information predicted stronger beliefs concerning the complexity and authenticity of animal emotions, as well as the moral relevance of animal emotions. This finding is consistent with previous literature that has described a link between experience with pets and beliefs about animal psychology [23,45,46]. Higher endorsement of the information source “My spiritual, moral, ethical, philosophical, or other belief systems” also predicted stronger belief in animal emotion complexity and authenticity, as well as animal emotion moral relevance. This novel finding will need to be replicated and studied more in future research to develop a better understanding of the effect. But it appears to be consistent with the idea that beliefs about the emotional experience and capacity of animals may depend upon one’s cultural context [19,24,55]. Unexpectedly, greater endorsement of podcasts as a source of information was associated with weaker beliefs concerning animal emotion complexity and authenticity. Because podcasts were the least-endorsed information source and the specific content of participants’ podcast exposure was not assessed (educational, entertainment, etc.), this finding should be interpreted cautiously. Future research will be needed to determine whether it reflects characteristics of podcast content, podcast listeners, or both.

### Potential animal welfare implications

Dietary and other consumption choices influence animal welfare, via both the number of animals used and the conditions under which they are kept [56,57]. Additionally, consumer (and voter) sentiment often drives corporate and policy shifts in animal welfare practices [58,59]. Given these connections between consumer behavior and animal welfare, it is important to develop a better understanding of the human psychology behind the choices people make in the marketplace [60,61]. The research described here demonstrates that the beliefs people hold about animal emotions are associated with self-reported animal-related behaviors, both dietary and non-dietary. This has potential implications for consumer-based interventions intended to impact animal welfare, although additional research will be required to make this connection more explicit.

### Study limitations

Several additional study limitations should be considered when interpreting and applying these results. All measures in the present studies relied on self-reported beliefs and behaviors. It has been argued that some beliefs are held implicitly, and may therefore be difficult to measure through methodology that depends on explicit reporting [62]. However, measuring implicit beliefs about emotion is notoriously difficult, and has so far eluded a large subfield of psychological scientists who study beliefs about human emotions [9]. Another concern with self-reported viewpoints and behaviors that have obvious ethical implications, such as those reported here, is the potential motivation to provide socially desirable responses. In the case of beliefs about animal emotions, scores on all three subscales of the BAES were previously shown to be uncorrelated to a measure of social desirability, suggesting that people are not simply providing responses that they think the researchers are seeking [13]. With regard to reported consumer behaviors, the rate of dietary avoidances reported in Study 1, which included an openly recruited sample, was very low, making it unlikely that participants were systematically inflating their self-reports. Further, as argued by Rosenfeld and Burrow [14], the dietarian-identity approach involves querying specific animal-product avoidances rather than self-applied labels (e.g., “vegan”), which should improve correspondence with actual consumption behaviors. Indeed, despite recruitment through a Prolific prescreen indicating prior self-identification as vegetarian or vegan, 30.7% of Study 2 participants reported no dietary exclusions. This discrepancy may reflect changes in dietary practices over time (i.e., since the Prolific pre-screens were filled out), or imperfect correspondence between self-applied dietary labels and current consumption patterns. Future work in this area may benefit from more direct measures of behavior including, for example, in-the-moment and in-the-field reports such as ecological momentary assessment [63].

Additional limitations pertain to sample characteristics. Both studies reported here were recruited from an online participant database, potentially limiting the generalizability of these findings to the broader population, although Prolific does at least appear to provide more representative samples compared to university student recruitment [29,30]. Nevertheless, both samples collected for this research were very highly educated compared to the general population of the U.S., where the studies were conducted. For example, the percentage of participants holding graduate degrees was 27% in Study 1 (open recruitment), and 41% in Study 2 (selective recruitment for vegans and vegetarians). This limitation is especially important to consider when interpreting the finding that increasing educational attainment was associated with less attribution of emotion function and experience to animals. Whether or not this finding also applies to high school educational attainment must await further research with more targeted recruitment. Of course, it will also be important and informative to expand this research to other geographical and cultural contexts in order to investigate and understand potential moderators of the relationship between beliefs about animal emotions and consumer behaviors that impact animals.

The present models included age, gender, and education as demographic covariates, but these variables do not capture the full range of factors likely to be related to animal-related avoidance behaviors. Such behaviors may be connected to other variables including but not limited to political orientation, religion, income, urban versus rural residence, pet ownership, other non-ethical reasons for food avoidances, and prior work or other direct experience with animals [15,64,65]. Some of these variables may also be associated with beliefs about animal emotions, raising the possibility that unmeasured third variables contributed to the observed associations. Accordingly, the present findings should not be interpreted as showing that beliefs about animal emotions alone explain animal-related avoidance behaviors. Rather, they show that BAES scores were associated with these behaviors above the demographic covariates included in the present models. Future research should examine whether these associations remain when broader ideological, experiential, socioeconomic, and motivational predictors are included.

## Conclusion

This research indicates that people’s beliefs about animal emotional capacity and experience are reliably associated with self-reported consumer behaviors that are related to animals. Further work is required to test causal pathways and to establish generalizability across cultural and geographical contexts beyond the US online samples studied here. But the consistency and magnitude of the associations observed suggest that these beliefs are a promising target for research on the potential determinants of consumer choices. If a causal connection can be demonstrated through additional research, then evidence-based communication about animal emotions may represent an approach to improving animal welfare indirectly through influence of consumer decision-making.

## Supporting information

S1 File S1-S5 Tables. This is Tables S1-S5.
(PDF)
