# Supplementary material for: Beliefs about animal emotions are associated with self-reported consumer behaviors related to animal use
Source: PLoS One. 2026 Jul 29;21(7):e0354794. doi: 10.1371/journal.pone.0354794 (PMC13419194; doi:10.1371/journal.pone.0354794)
Supplement: S1 File — This is Tables S1-S5. (PDF) [file pone.0354794.s001.pdf]

Supplemental Materials: Tables S1-S5

Table S1 – *Study 1: Regressions of Associations Between Beliefs about Animal Emotions and Demographic Variables*

Variable	*B*	*SE B*	β	*t*	*p*	R^2
BAES 1						0.056***
Constant	4.091***	0.331		12.365	<0.001	
Age	0.013*	0.005	**0.129**	2.391	0.017	
Gender	0.296*	0.150	**0.106**	1.980	0.048	
Bachelors	-0.389*	0.177	**-0.138**	-2.195	0.029	
Graduate	-0.610**	0.197	**-0.194**	-3.096	0.002	
BAES 2						0.011
Constant	3.142***	.286		10.977	<.001	
Age	-.004	.005	-.045	-.808	.420	
Gender	.206	.129	.087	1.594	.112	
Bachelors	-.108	.153	-.045	-.703	.482	
Graduate	-.157	.170	-.059	-.924	.356	
BAES 3						0.047**
Constant	3.769***	0.347		10.859	<0.001	
Age	-0.004	0.006	-0.037	-0.681	0.496	
Gender	0.633***	0.157	**0.217**	4.030	<0.001	
Bachelors	-0.136	0.186	-0.046	-0.730	0.466	
Graduate	-0.152	0.207	-0.047	-0.738	0.461	

Note. Criterion (outcome) variables for this analysis were Belief about Animal Emotions (BAES) subscale scores for Study 1, *N* = 340 (only Man and Woman genders) from open recruitment on Prolific.com. *Bachelors*, highest degree attainment bachelors or equivalent. *Graduate*, highest degree attainment graduate-level. *BAES 1*, beliefs about animal emotion complexity and authenticity; *BAES 2*, beliefs regarding independence of animal emotions from human similarity; *BAES 3*, beliefs about moral relevance of animal emotions. Interpretable standardized beta-weights are bolded for emphasis.
*$p < 0.05$; **$p < 0.01$; ***$p < 0.001$.

Table S2 – *Item-Level Response Rates to Exploratory, Forced-Choice Item Regarding Sources of Beliefs about Animal Emotions, Study 1*

Item	#	%
educational classes (high school, college, etc.)	22	6.4%
things my pets do (past and present)	170	49.6%
behavior of animals I work with (job, farm, etc.)	34	9.9%
social media videos (tik tok, facebook, etc.)	14	4.1%
documentary movies and tv	71	20.7%
fictional stories, folk tales, or cultural stories about animals	11	3.2%
other (please specify)	21	6.1%

Note. For Study 1, *N* = 343 from open recruitment on Prolific.com. *#,* number of participants that endorsed each item as having had "the most influence on your beliefs about the emotional lives of animals"; *%,* percentage of the sample that endorsed each item. As explained in the main text, these values should be interpreted with caution because participants were only able to select a single source. An updated sources of beliefs measure was developed for Study 2.

Table S3 – *Verbatim narrative responses for "other" belief sources, Study 1*

1. My philosophy of the science of human/animal behavior, radical behaviorism
2. Reading
3. My own varied experiences in life and connection to nature
4. Observing animal behaviors over the years
5. My parents beliefs
6. I've done some limited research on this topic out of my own curiosity
7. A Mix of classes, documentaries, social media videos and my pets.
8. My spiritual belief that all living things deserve to be treated kindly
9. Personal observations and experiences based on the animals I have encountered
10. My upbringing in rural America and connection to the outdoors
11. Interactions with animals in nature
12. podcasts
13. Farm life and God's structure of creation
14. The Bible
15. Research into animals and their behavior (I follow a plant based diet)
16. Psychology books
17. Personal interest, I've looked into this and read about it in various sources for laypeople, like Scientific American or NatGeo or Discovery, etc.
18. things other people's pets do
19. My own moral and ethical values
20. Podcasts like ologies and other forms of content that get information from scientific experts
21. The Bible, the way Jesus talks about shepards and sheep, a wondering of what Adam and Eve's relationship with animals was like before sin.

Table S4 – *Study 2: Regressions of Associations Between Beliefs about Animal Emotions and Demographic Variables*

Variable	*B*	*SE B*	β	*t*	*p*	R^2
BAES 1						0.126***
Constant	5.359***	0.416		12.879	<0.001	
Age	0.011	0.006	0.100	1.632	0.104	
Gender	0.016	0.171	0.006	0.094	0.925	
Bachelors	-0.945***	0.231	**-0.340**	-4.093	<.001	
Graduate	-1.258***	0.227	**-0.455**	-5.544	<.001	
BAES 2						0.083***
Constant	3.658***	0.384		9.524	<.0001	
Age	0.009	0.006	0.091	1.457	0.146	
Gender	-0.005	0.158	-0.002	-0.031	0.975	
Bachelors	-0.444	0.213	-0.177	-2.082	0.038	
Graduate	-0.899***	0.210	**-0.361**	-4.292	<0.001	
BAES 3						0.119**
Constant	4.947***	0.425		11.646	<.001	
Age	0.005	0.007	0.044	0.717	0.474	
Gender	0.341	0.174	0.119	1.952	0.052	
Bachelors	-0.838***	0.236	**-0.296**	-3.555	<.001	
Graduate	-1.210***	0.232	**-0.430**	-5.222	<.001	

Note. Criterion (outcome) variables for this analysis were Belief about Animal Emotions (BAES) subscale scores for Study 2, *N* = 247 (only Man and Woman genders) from targeted recruitment for vegans and vegetarians on Prolific.com. *Bachelors*, highest degree attainment bachelors or equivalent. *Graduate*, highest degree attainment graduate-level. *BAES 1*, beliefs about animal emotion complexity and authenticity; *BAES 2*, beliefs regarding independence of animal emotions from human similarity; *BAES 3*, beliefs about moral relevance of animal emotions. Interpretable standardized beta-weights are bolded for emphasis.
*$p < 0.05$; **$p < 0.01$; ***$p < 0.001$.

Table S5 –*Regressions of Associations Between Beliefs about Animal Emotions and Belief Sources*

Variable	*B*	*SE B*	β	*t*	*p*	R^2
BAES 1						0.304***
Constant	4.319***	.643		6.722	<.001	
Age	.004	.006	.039	.670	.504	
Gender	.047	.157	.017	.298	.766	
Bachelors	-.405	.229	-.146	-1.768	.078	
Graduate	-.525*	.242	**-.190**	-2.166	.031	
Doc mov/tv	.125	.136	.058	.920	.359	
Social media	-.188	.119	-.102	-1.582	.115	
Pets	.457*	.181	**.163**	2.531	.012	
Work	-.080	.111	-.043	-.717	.474	
Edu. Classes	-.177	.123	-.097	-1.445	.150	
Reading	.020	.128	.010	.160	.873	
Podcasts	-.535***	.130	**-.268**	-4.117	<.001	
Belief system	.424***	.115	**.219**	3.697	<.001	
BAES 2						0.116**
Constant	4.019***	.653		6.159	<.001	
Age	.008	.006	.087	1.334	.183	
Gender	-.008	.160	-.003	-.049	.961	
Bachelors	-.312	.233	-.124	-1.338	.182	
Graduate	-.748**	.246	**-.300**	-3.040	.003	
Doc mov/tv	.080	.138	.041	.578	.564	
Social media	.093	.120	.056	.773	.440	
Pets	-.145	.184	-.057	-.790	.430	
Work	-.180	.113	-.109	-1.599	.111	
Edu. Classes	-.146	.124	-.089	-1.172	.242	
Reading	.048	.130	.027	.373	.709	
Podcasts	-.098	.132	-.054	-.742	.459	
Belief system	.147	.116	.084	1.264	.207	
BAES 3						0.210***
Constant	3.347***	.696		4.808	<.001	
Age	.002	.007	.020	.327	.744	
Gender	.315	.170	.110	1.849	.066	
Bachelors	-.511*	.249	**-.181**	-2.056	.041	
Graduate	-.781**	.263	**-.278**	-2.974	.003	
Doc mov/tv	.064	.147	.029	.439	.661	
Social media	.032	.129	.017	.250	.803	
Pets	.439*	.196	**.154**	2.241	.026	
Work	-.082	.120	-.044	-.678	.498	
Edu. Classes	-.199	.133	-.108	-1.502	.134	
Reading	-.071	.138	-.035	-.511	.610	
Podcasts	-.157	.141	-.077	-1.113	.267	
Belief system	.448***	.124	**.227**	3.611	<.001	

Note. Criterion (outcome) variables for this analysis were Belief about Animal Emotions (BAES) subscale scores for Study 2, N = 247 (only Man and Woman genders) from targeted recruitment for vegans and vegetarians on Prolific. *Bachelors*, highest degree attainment bachelors or

equivalent. *Graduate*, highest degree attainment graduate-level. *BAES 1*, beliefs about animal emotion complexity and authenticity; *BAES 2*, beliefs regarding independence of animal emotions from human similarity; *BAES 3*, beliefs about moral relevance of animal emotions. Interpretable standardized beta-weights are bolded for emphasis.
*$p < 0.05$; **$p < 0.01$; ***$p < 0.001$.
